# Leveraging deep contrastive learning for semantic interaction

**DOI:** 10.7717/peerj-cs.925

**Published:** 2022-04-08

**Authors:** Mahdi Belcaid, Alberto Gonzalez Martinez, Jason Leigh

**Affiliations:** 1 University of Hawaii at Manoa, University of Hawaii at Manoa, Honolulu, HI, United States; 2 University of Hawaii at Manoa, Laboratory for Advanced Visualization and Applications, Honolulu, Hawaii, United States

**Keywords:** Semantic interaction, Deep learning, Visual analytics, Natural language processing

## Abstract

The semantic interaction process seeks to elicit a user’s mental model as they interact with and query visualizations during a sense-making activity. Semantic interaction enables the development of computational models that capture user intent and anticipate user actions. Deep learning is proving to be highly effective for learning complex functions and is, therefore, a compelling tool for encoding a user’s mental model. In this paper, we show that deep contrastive learning significantly enhances semantic interaction in visual analytics systems. Our approach does so by allowing users to explore alternative arrangements of their data while simultaneously training a parametric algorithm to learn their evolving mental model. As an example of the efficacy of our approach, we deployed our model in Z-Explorer, a visual analytics extension to the widely used Zotero document management system. The user study demonstrates that this flexible approach effectively captures users’ mental data models without explicit hyperparameter tuning or even requiring prior machine learning expertise.

## Introduction

The proliferation of data far outpaces our ability to analyze it. Using fully autonomous machine learning methods for data analysis is difficult in applications such as clustering, where domain knowledge and subjective cluster preferences are challenging to encode. Human-in-the-loop machine learning has emerged as a great paradigm to combine a user’s insight into model inference. Human-in-the-loop models can be steered and tuned within a tight loop in response to changing perspectives, preferences, or to take into account domain knowledge. Within visual analytics systems, semantic interaction (SI) is the human-in-the-loop machine learning approach that aims to empower users to manipulate the internal parameters of a parametric projection algorithm solely *via* interactions with a 2D visualization (Bradel, North & House, 2014; Dowling et al., 2018b; Sacha et al., 2016). To do so, SI systems learn to translate user interaction (in our case users re-position points on the 2D canvas), as users re-position points on the 2D canvas, into valuable training information. SI systems most often accomplish this objective by inverting their models to transform user input (changes to the layout) into parametric feedback (updates to the input); this process is known as model steering.

Methods such as t-distributed stochastic neighbor embedding (t-SNE) (Van der Maaten & Hinton, 2008) and uniform manifold approximation and projection (UMAP) (Becht et al., 2019) are two of the most popular techniques for visualizing higher dimensional data in a 2D space and for clustering in visual analytics. UMAP finds a lower-dimensional representation of data with similar topological properties as the high dimensional space by measuring the distance of points across a neighborhood graph of the high dimensional data and therefore using optimization to find the closest topological structure in a lower-dimensional space (McInnes, Healy & Melville, 2018). Despite UMAP’s and t-SNE’s efficacy, SI systems typically use simple and interpretable linear models, such as LDA (Latent Dirichlet Allocation) (Blei, Ng & Jordan, 2003) and PCA (Principal Component Analysis) (Abdi & Williams, 2010), to deliver a bidirectional pipeline that can project a document’s feature representation on a canvas, and update the model parameters based on how an analyst repositions a document, thus incorporating their intent in the model. This bidirectionality is particularly difficult to implement in algorithms such as t-SNE and UMAP, since these are not invertible, meaning that they cannot seamlessly transform user input (changes to the layout) into parametric feedback (Espadoto, Hirata & Telea, 2020).

In spite of their advantages, deep learning algorithms are difficult to optimize without considerable expertise or extensive annotated datasets (LeCun, Bengio & Hinton, 2015). Self-supervised learning has recently emerged as a promising method to train deep learning models by relying on data generated from the raw input, thus circumventing the time-consuming and costly process of data annotation. A self-supervised approach, contrastive learning uses small transformations on raw data to create training data that is then used to maximize the similarities of positive pairs (*e.g*., documents about similar topics) while minimizing the similarities of negative ones Chen et al. (2020). We use this idea to generate training data from documents laid out by a user on a canvas and leverage this data to train a model to layout new documents in a way that reflects the user’s mental model.

In this paper, we propose a method that combines SI with contrastive models to allow non-experts to guide deep learning by using contrastive models to mimic state-of-the-art dimensionality reduction methods. This paper will begin by introducing prior work in SI, emphasizing the need and challenges to provide functions to not only project data onto a 2D canvas, but also an inverse function to transform the input following updates the layout on the canvas (“Basic Assumptions of the Model”). We will also review work in contrastive models to show how their properties make them useful in SI systems (“Addition of an Embedding Mean Square Error Objective”). With that grounding, we will then explain our contrastive model’s approach in SI (“Z-Explorer User Study”) and describe a specific implementation of our approach in Z-Explorer, a custom tool for the visual exploration of document collections. Finally, we will conclude with a summary of the findings of our user study.

## Related Work

### Semantic interaction pipelines: the inverse computation problem

**Figure 1:**
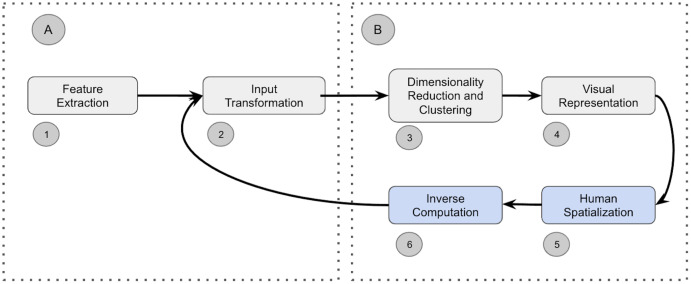
(A–B) Sense-making loop and steps comprising a visual analytics pipeline.

Visual analytics workflows (Endert, Bradel & North, 2013; Shneiderman, 2003; Moreland, 2012; Endert, 2014) use an iterative process that mirrors the steps present in the sense-making loop of Pirolli & Card (2005) to transform raw data into visualizations. Existing visual analytics pipelines have converged towards a general pipeline that comprises the following four primary steps (Cao & Cui, 2016) depicted in Fig. 1: (1) feature extraction, (2) input transformation, (3) dimensionality reduction and clustering, and (4) visual representation. The first block (A) focuses on converting raw data into vectors suitable for machine learning. The resulting abstract vectors are subsequently transformed in the second block (B) into human interpretable representations.

In semantic interaction (Endert, 2014; Bradel, North & House, 2014; Jeong et al., 2009) users manipulate the system by intuitively interacting with the visual representation of the data (such as clicking on data points and re-positioning them). These natural interactions allow users to remain in their cognitive zone and enhance their analysis efficiency (Wenskovitch & North, 2017). In SI, users generate a new spatialization (block 5 in Fig. 1) that forces the underlying data analytics algorithm to learn new parameters to semantically interpret these interactions. As depicted in block 6, existing SI systems commonly need to invert the computation of the underlying pipeline to enable the user’s semantic interactions to be communicated to the model as parametric feedback (arrow between blocks 6 and 2). Parametric feedback describes the mechanism used to adjust a model’s parameters based on a user’s abstract interactions with the visual representation. The SI paradigm does not specify how parametric feedback should be computed. This turns out to be one of the major limitations of the general approach for semantic interaction (Leman et al., 2013). While visual analytics often uses efficient but uninvertible “black-box” dimensionality reduction and clustering methods like t-SNE (Van der Maaten & Hinton, 2008), UMAP (McInnes, Healy & Melville, 2018), or H-DBSCAN (McInnes, Healy & Astels, 2017), SI systems rely principally on rudimentary models (Leman et al., 2013; Self et al., 2015; House, Leman & Han, 2015; Sacha et al., 2016; Dowling et al., 2018a) for which an inverse computation, and, hence, parametric feedback, can be easily derived.

Recent work described in (Bian & North, 2021a, González Martinez et al., 2020a) argues that the ability of SI systems to infer an analysts’ precise intent depends on the underlying data representation (quadrant A in Fig. 1). This work showcased the use of Deep Learning for the Feature Extraction and Transformation steps of the pipeline. The work presented in (González Martinez et al., 2020b) focuses on the parametric feedback by proposing a surrogate model to transverse the dimensionality reduction, clustering, and visual representation steps of the pipeline (quadrant B). This paper extends the latter approach by demonstrating how contrastive learning is a more effective approach for realizing the potential of SI.

### Contrastive learning

Deep Contrastive Learning (DCL) is an efficient method for learning desirable embeddings of high-dimensional data (Chen et al., 2020; Giorgi et al., 2020; Khosla et al., 2020; Li et al., 2020; Arisdakessian et al., 2021). Consider, for example, a siamese deep neural network architecture, which consists of two identical artificial neural networks (feedforward perceptrons) that work in parallel to learn the hidden semantic similarity between the projected representations of a pair of input vectors (Bromley et al., 1993; Chicco, 2021). Combined with a contrastive optimization objective, siamese neural networks can learn a nonlinear function to map high dimensional input data to a low dimensional representation (Hadsell, Chopra & LeCun, 2006).

Models using DCL do not require an explicit similarity metric to be computed on the entire input dataset. Compared to Weighted Multi-Dimensional Scaling (WMDS) (Young & Harris, 1983), which are commonly used in SI, DCL provides two clear benefits: First, it allows the model to be trained on subsets of the data, thereby reducing complexity and enabling the close to real-time processing speeds needed in these human-in-the-loop systems (González Martinez et al., 2020a). Second, DCL enables categorical relationships to be learned on the fly based on the user’s domain knowledge, communicated to the model by manipulations of a data visualization. The ability of DCL models to learn complicated nonlinear input transformations and to map out-of-sample data efficiently makes them ideal for capturing users’ mental models as they interact with data. Lastly, the smooth and coherent mapping generated by the function makes this modeling approach especially attractive to apply at the last step of a SI pipeline for obtaining more interpretable visualizations.

Existing research has shown that DCL is an effective method for clustering data. For example, our previous work showed that DCL could attain 94% clustering accuracy on the BBC News articles dataset (González Martinez et al., 2020a). More broadly, neural networks can efficiently mimic the embeddings of various dimensionality reduction algorithms, including UMAP (Espadoto, Hirata & Telea, 2020). According to Zhang et al. (2021), DCL outperforms state-of-the-art approaches on eight key benchmark datasets, including Google News and Twitter datasets, which cover 152 and 89 categories, respectively. Specifically, the authors report that DCL can yield clustering accuracy results up to 11% better than alternative methods on short texts, as well as better intra- and inter-cluster distances, which are crucial in semantic interaction.

## Proposed Approach: Contrastive Learning for Semantic Interaction

We will first elaborate on the basic assumptions of our model (“Participants and Experimental Dataset”). Then we will describe how we maintain the positions of the clustered documents in the visualization space as the DCL is fed new semantic interaction input (“Procedure”).

### Basic assumptions of the model

The model described herein borrows heavily from that described in Hadsell, Chopra & LeCun (2006), Wooton (2020). The general architecture of a Siamese neural network employing a contrastive loss function can be formally specified as follows: given a labeled dataset of *N* input/output pairs, wherein each input is a *D*-dimensional vector of real-valued features, and each output is one of the k-class labels, the goal is to learn a parametric dimensionality reduction function with $d\; {\ll} \;D$ and with the following properties:

1. For any two inputs with the same output label, the Euclidean distance in lower-dimensional space between the reduced outputs is as close to 0 as possible.
2. For any two inputs with different output labels, the Euclidean distance in lower-dimensional space between the reduced outputs is as close as possible to a chosen margin *m* > 0.

Ideally, the transformation function should be differentiable with respect to the set of parameters (*W*) of the DCL, allowing backpropagation with gradient descent to be used for finding the optimal parameters *W* satisfying constraints 1 and 2 above. We assume that the high dimensional input representations of two documents ${x_i}$ and ${x_j}$ that belong to the same class *y* are similar to one another based on some abstract notion of similarity perceived by the annotator. That is, two points ${x_i}$ and ${x_j}$ are similar if and only if their labels ${y_i}$ and ${y_j}$ are identical. In an interactive human-in-the-loop context (such as shown from our user study discussed below), the expertise of a human analyst can be leveraged on-the-fly to provide such a labelling when labels ${y_i}$ are unavailable. This labeling is entirely dependent upon the human analyst’s understanding of the relationships present in the data. The margin *m* can be understood as a scaling factor and should be chosen based on the desired level of spatial distance between disparate points in the embedding space. In this paper, we chose *m* = 1 to prevent the scale of the embedding space from growing arbitrarily large.

Given a labeled dataset and a chosen margin *m*, a corresponding contrastive dataset

$C = {{{\{}(x_i,x_j, r_i, r_j, y_i,y_j,c)_p }{\}}_P}$ is produced such that:

(1) $${c_p} = \left\{ {\matrix{ 0 \hfill & {{\rm if}\;{y_{ip}} = {y_{jp}}} \hfill \cr m \hfill & {{\rm otherwise}} \hfill \cr } } \right.$$

with *c*_*p*_ representing the target Euclidean distance between inputs *x*_*ip*_, *x*_*jp*_, and 1 *≤ p ≤ P ≤ N*^2^ where *P* represents the number of unique pairs in the contrastive dataset. Initial values of the target Euclidean distance (*c*_*p*_) can be computed between documents that were randomly laid out on the canvas, or that were arranged using a lower-dimensional representation of the data (*e.g*., using UMAP). The set of pairwise-distance between documents is re-computed in subsequent iterations using the document layout created by the user. Note that we also include the reduced representations *r*_*i*_, *r*_*j*_ and class labels *y*_*i*_, *y*_*j*_ as outputs in each contrastive seven-tuple. Including the class label *y* was motivated by the need to capture the cluster labels applied by the user when organizing the data visually with semantic interaction. The motivation for including the reduced points *r* as outputs is more subtle and will be explained in “Addition of an Embedding Mean Square Error Objective”.

**Figure 2:**
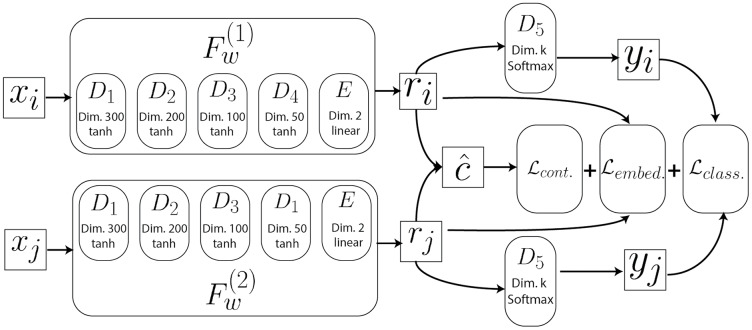
Architecture of the neural network. See “Deep Learning Model” for more details.

The full model architecture used in this study is illustrated in Fig. 2 and further described in “Deep Learning Model” and in Wooton (2020). This model contains twin copies of a feed-forward neural network *F*_*w*_, with shared weights *W*, each of which operate on one of the two input vectors *x* in a contrastive tuple (*x*_*i*_, *x*_*j*_, *r*_*i*_, *r*_*j*_, *y*_*i*_, *y*_*j*_, *c*)_*p*_ (See “Deep Learning Model” for more details). For a given input pair (*x*_*i*_, *x*_*j*_)_*p*_ this model first produces a pair of points of reduced dimensionality (*r*_*i*_, *r*_*j*_)_*p*_ using the learned mapping *F*_*w*_ encoded in each of the twin networks. A prediction of the target distance ${\hat c_p} = ||{r_i}p - {r_j}p{||_2}$ is then computed on these reduced points. Additionally, a softmax cluster label prediction *y*_*i*_, *y*_*j*_ is also computed by passing the reduced representations *r* to a *k*-dimensional softmax layer. Given this specification, the task of finding the optimal parameters *W* of *F*_*w*_ involves minimizing the following loss function:

${{\rm \cal{L}}_{total}} = {{\rm \cal{L}}_{contrastive}} + {{\rm \cal{L}}_{classifier}} + {{\rm \cal{L}}_{embedding}})$

The three component terms of this total loss are defined by the following equations:

${{\rm \cal{L}}_{contrastive}} = MSE = \displaystyle{1 \over P}\sum\limits_{p = 1} P {({c_p} - {\hat c_p})^2}$

${{\rm \cal{L}}_{classifier}} = \displaystyle{1 \over P}\sum\limits_{p = 1}^P {\sum\limits_k {({y_{ik}}log(} } \hat yik) + {y_{jk}}log(\hat yjk){)_p}$

${{\rm \cal{L}}_{embedding}} = \displaystyle{1 \over P}\sum\limits_{p = 1}^P {{{({r_{ip}} - {{\hat r}_{ip}})}^2}} + {({r_{jp}} - {\hat r_{jp}})^2}$

In order to fine-tune the contribution of each of these terms to *L*_*total*_, weight hyperparameters *α*, *β*, and *γ* were introduced to adjust the loss contribution, yielding a weighted loss function of:

${{\rm \cal{L}}_{total}} = \alpha \cdot {{\rm \cal{L}}_{contrastive}} + \beta {{\rm \cal{L}}_{classifier}} + \gamma {{\rm \cal{L}}_{embedding}})$

In our user study, parameter values of *α* = 0.25, *β* = 0.25, and *γ* = 0.5 were chosen such that the embedding loss would account for 50% of *L*_*total*_.

### Addition of an embedding mean square error objective

**Figure 3:**
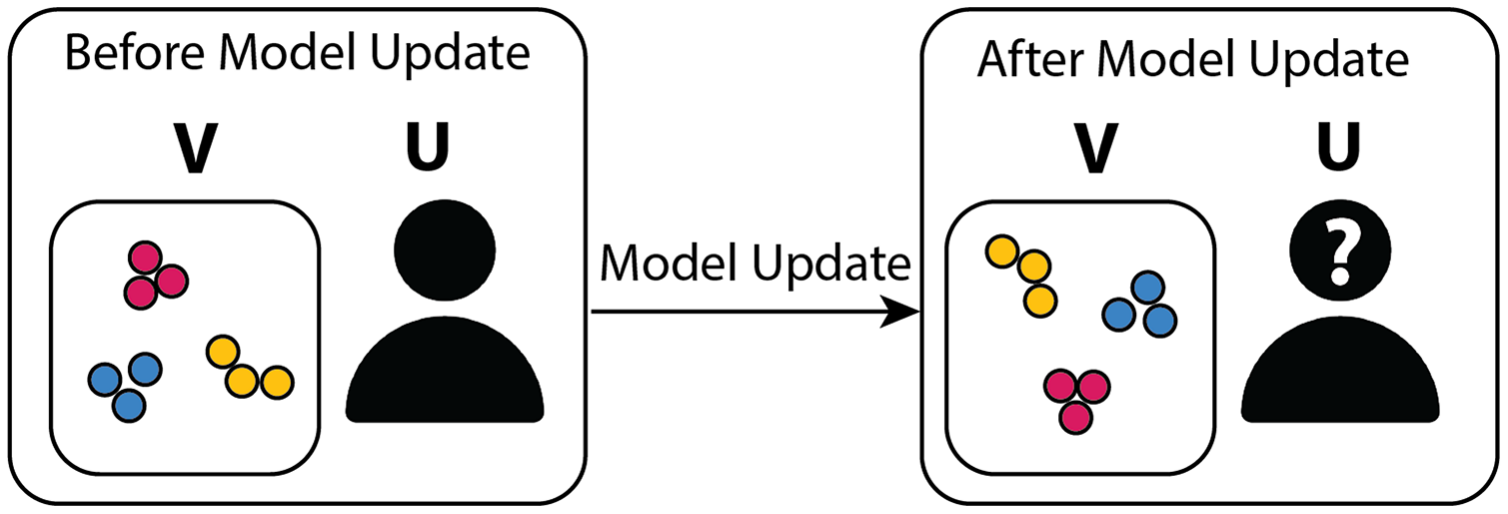
Graphical diagram summarizing the motivation behind adding a position-based embedding objective to the model. On the left-hand side, the user ${\rm U}$ organized the points in the visualization V into separate clusters of blue, red, and green points and decides to initiate a model update cycle. The model parameters are then adjusted based on the user feedback. In the absence of L*embedding*, the model keeps the blue, red, and green points well clustered in the updated visualization (right-hand side) but fails to maintain the relative cluster positions (the blue points have migrated to the top half of the visualization, and the relative left-right ordering of the green and red clusters has been flipped), thus perturbing the user’s mental model, particularly in the presence of a large number of clusters, and leading to confusion.

As mentioned previously, the inclusion of the embeddings *r* as model outputs is subtle, and the motivation for doing so requires clarification. In an interactive setting, deep contrastive learning has the additional key requirement of maintaining the relative inter-cluster positions specified by the user between model updates. Since the contrastive loss does not determine how the function *F*_*w*_ positions clusters in the visual space, the inter-cluster layout specified by the user may change following model updates, as illustrated in Fig. 3.

In order to avoid cluster positions being lost between model updates, an embedding loss *L*_*embedding*_ is added to the model. As a consequence, embeddings that differ substantially from the original embeddings are penalized. Specifically, contrastive learning is applied to the dataset containing *r* = (*r*_*i*_, *r*_*j*_), the user’s original 2D positions, and embeddings /*hat*[*r*] that minimize *L*_*[embedding]*_ are predicted for every input *x*.

It may be difficult for the model to optimize both *L*_*contrastive*_ and *L*_*embedding*_ simultaneously when the points in clusters are close together. Nevertheless, we assume here that the users’ motivation is to organize data, *i.e*., to group similar documents and to separate out those that aren’t. Therefore, it is reasonable to assume that clusters would be spatially separated in a manner that wouldn’t impede the simultaneous optimization of *L*_*contrastive*_ and *L*_*embedding*_.

After reorganizing the visual space, the user submits the newly labeled dataset to fine-tune the model inferred from previous steps. The training is described in See “Deep Learning Model”.

Since there are currently no existing feedback mechanisms for updating UMAP parameters based on user interactions, a comparison between the utility of UMAP and DCL in SI was not necessary.

## Z-Explorer User Study

**Figure 4:**
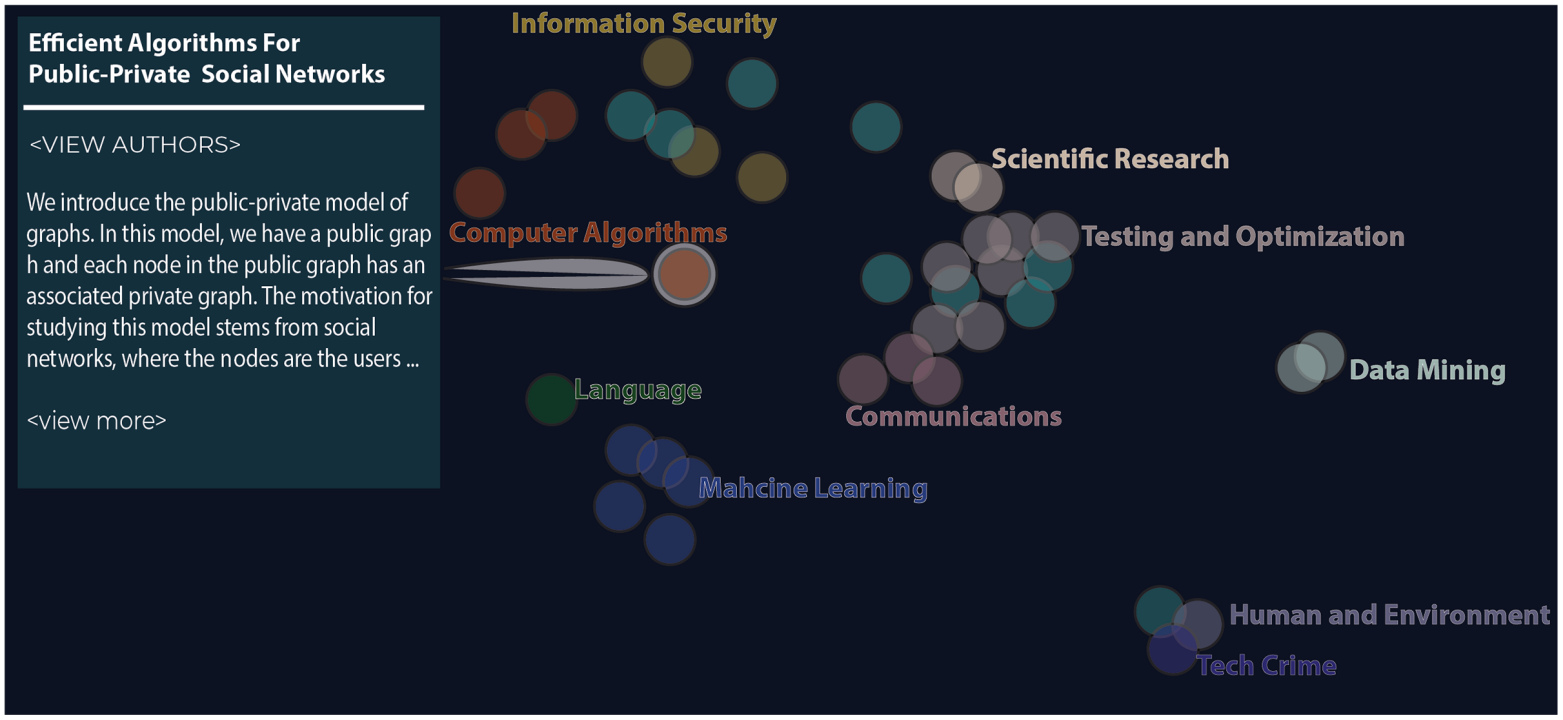
The figure shows the Z-Explorer interface at an intermediate step in one of the studies. The interface consists of an interactive scatter plot where each paper is encoded as a circle. Similar documents are closer that distant ones. Users can drag individual documents and assign labels to create custom groupings like those shown in the figure. Participants can browse a document’s abstract by hovering over its circle.

To evaluate the efficacy of our model to end-users, we designed and performed a user study using Z-Explorer- our visual analytics tool plug-in for the Zotero reference management software (González Martinez et al., 2020a) (Fig. 4-picture of actual Z-Explorer). Z-Explorer uses the contrastive learning-based SI approach described above to let users interactively refine existing clusters of documents (represented as dots in a scatter plot). These user-initiated updates, *i.e*., semantic interactions, inform our model and enable new documents to be clustered in a way that best supports a user’s mental model.

Using Z-Explorer, we conducted a study to answer two questions:

- Q1: Is the deep contrastive method capable of spatially organizing the documents in a way that is congruent with the user’s mental model? Answering this question will allow us to validate the efficacy of DCL in leveraging user interaction with the data to capture their mental model.
- Q2: How does the goodness of clustering compare between Z-Explorer and UMAP in a steerable context? This question was designed to assess whether using UMAP, a state-of-the-art projection algorithm, in a supervised way to learn from the users’ cluster arrangements prior to predicting documents’ positions would outperform the approach implemented in Z-Explorer.
- Q3: Do users manually cluster documents into similar or distinct clusters? We hypothesize that manually clustering a set of documents is a subjectivity activity and that a users’ experience may determine how they cluster a set of documents. In that case, human-in-the-loop is beneficial for clustering, since it allows users to determine how data should be clustered without doing the work of manually clustering it.

### Participants and experimental dataset

Fourteen computer science students (Five PhDs, Seven Masters and Two undergraduates) participated in the study. The experimental dataset comprised 52 abstracts from computer science papers (These papers won Best Paper awards at the CHI, AAAI, SIGCOMM, SP, KDD conferences since 1996) (Huang, 2020). This dataset covers a diversity of computer science topics, making it ideal for answering Question Q2, *i.e*., whether users’ computational background would yield similar or distinct clusters for the same documents. The University of Hawaii at M $\bar {\rm a}$noa granted ethical approval to conduct this study within its facilities (application # 2020-00412). All participants in our study provided written informed consent.

### Procedure

The abstracts were converted intro vectors suitable for machine learning using the BERT model. Specifically, the BERT model (Devlin et al., 2018) was used to sequentially encode a sentence’s input tokens. The hidden state of the CLS token added by the BERT WordPiece Tokenizer was used as the sentence embedding Feng et al. (2020). A paragraph’s embedding was calculated simply by averaging its sentences’ embeddings.

Due to COVID-19 restrictions, all studies were conducted *via* recorded video conferencing sessions (using Zoom). To establish an initial baseline, every user was given an arrangement of documents. In order to avoid influencing the users’ perception of how documents should be arranged, the initial set of documents has been placed randomly on the canvas. The decision was communicated to users.

**Figure 5:**
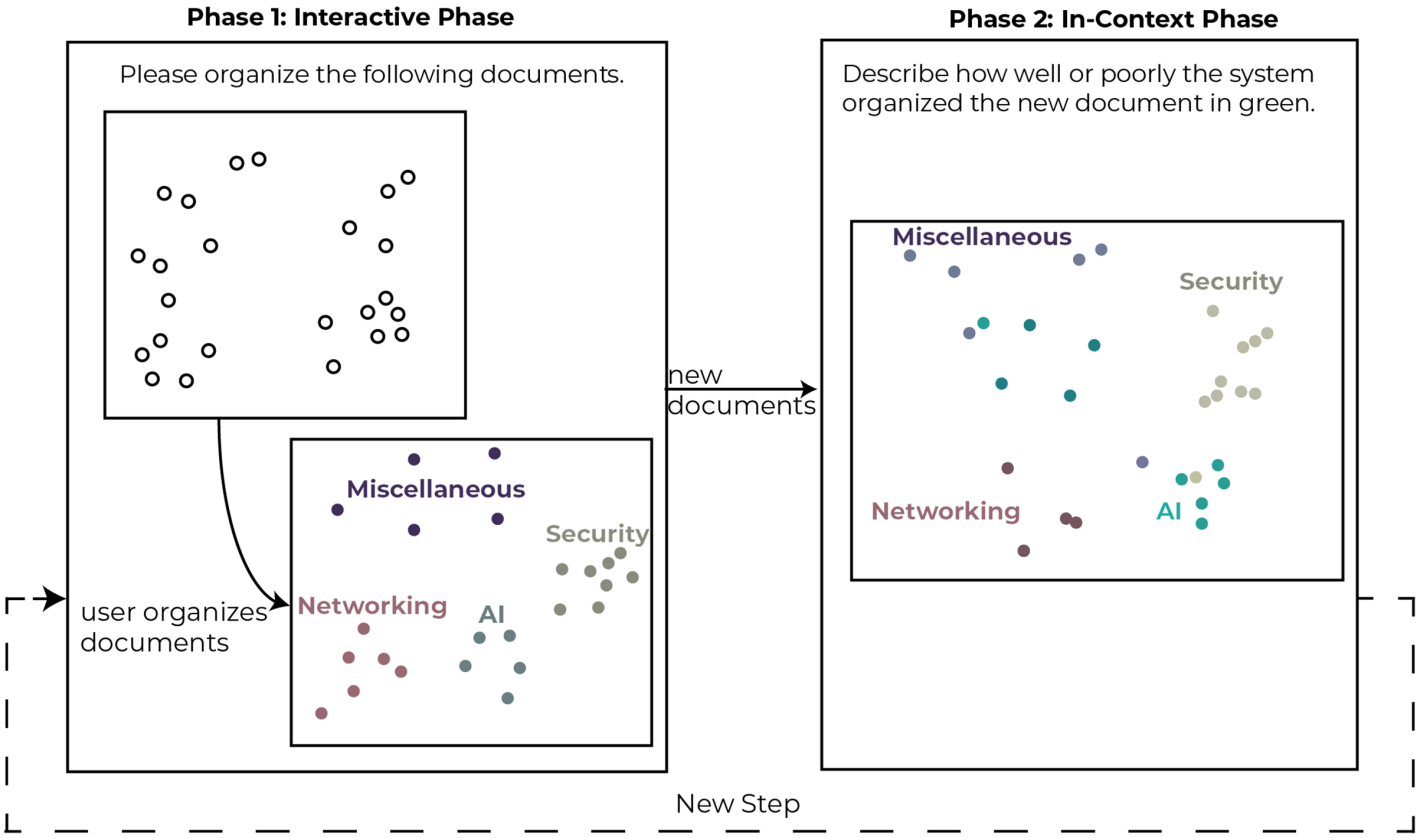
Depiction of the phases of a single step in the study. This two-phase process was completed up to 5 times with each participant.

In each step of the study, users were asked to use Z-Explorer to read and organize a subset of the documents represented as 2D points in a scatter plot. Then over five additional steps, documents were introduced in batches of ten documents at a time. Each step consisted of two phases (see Fig. 5). In the first phase (Interactive Phase), participants were asked to manually re-cluster the documents (if necessary) in the scatter plot. We did not restrict the number of clusters the participants could create, including allowing them to create single document clusters. The interactions performed in this phase were interpreted by Z-Explorer to create its internal representation of the user’s mental model. In the second phase (In-Context Phase), new documents were added to the scatter plot and automatically clustered based on Z-Explorer’s learned mental model of the user.

Participants were then asked to review the newly introduced documents and describe how well or poorly the system performed the new clustering. We repeated this procedure five times, adding different sets of documents at each step until all 52 papers were incorporated. On average, users took 60 minutes to complete the study, and due to time restrictions, five users did not complete all five steps.

### Data collected

To answer our three research questions, our research study focused on collecting both quantitative and qualitative survey data. Specifically, to answer question Q1 (whether the approach presented here is capable of learning the users’ mental model), we recorded the individual document movements per step of the study to show how well our model performed when positioning the new documents in each step. We enhanced our quantitative data collection with qualitative data collection where participants were asked to explain why they chose to create or modify clusters and why they chose to reposition or not reposition specific documents.

We compared Z-Explorer to UMAP in order to answer Q2 and determine whether steerable models are advantageous for users compared with a state-of-the-art dimensionality reduction technique such as UMAP. Although the UMAP algorithm is primarily used for unsupervised dimension reduction, its significant flexibility allows it to easily be extended to other tasks such as supervised dimensionality reduction and metric learning (?). To mimic a steerable context, we benchmarked Z-Explorer’s goodness of clustering against that of UMAP in supervised learning mode.

To answer Q3 (whether users would create different groupings in the same dataset), we recorded the number of clusters created in each study and the users’ document to cluster assignments, which were compared across users and steps. To ensure a fair comparison, we only used the data from the eight study participants who examined all 52 documents and for whom the document-to-cluster assignments were recorded. We calculated the V-measure score, an entropy-based external cluster evaluation used to measure homogeneity and completeness (Rosenberg & Hirschberg, 2007), across the eligible studies to determine the clusters’ similarity across users. The data is only shown for nine of the participants. See “Model Training” for more information.

### Computational analysis

The model was implemented using TensorFlow (Abadi et al., 2015) in Python, while the user interface was implemented using the Angular JavaScript framework (Jain, Bhansali & Mehta, 2014). In each iteration, the model is retrained with all the data to minimize the intra-cluster distances between points while maintaining the inter-cluster distance across documents as close to the margin value as possible (‘m’ = 1). To minimize the wait time between updates, the model is fitted for 10 epochs using the Adam optimizer with a learning rate of *α* = 0.1. The server implementation of the model (SageBrain) and the accompanying user interface (zotAngular) are provided as a reference at linked to from the following GitHub repository (https://github.com/whatwehaveunlearned/zExplorer).

## Results and Discussion

### Q1: Does the contrastive learning-based model faithfully represent the user’s mental model?

Our hypothesis was that the model trained on the users’ interactions would reflect the users’ mental model by positioning new documents in a manner that is consistent with their groupings. This can be measured by either 1-tracking the number of document movements, which we assume should be low if the model accurately predicts cluster assignments, by 2-comparing the difference between the model-generated cluster predictions and user-generated cluster predictions at each step or 3-by explicitly asking the users to rate the system. The results for these three queries are described below.

**Figure 6:**
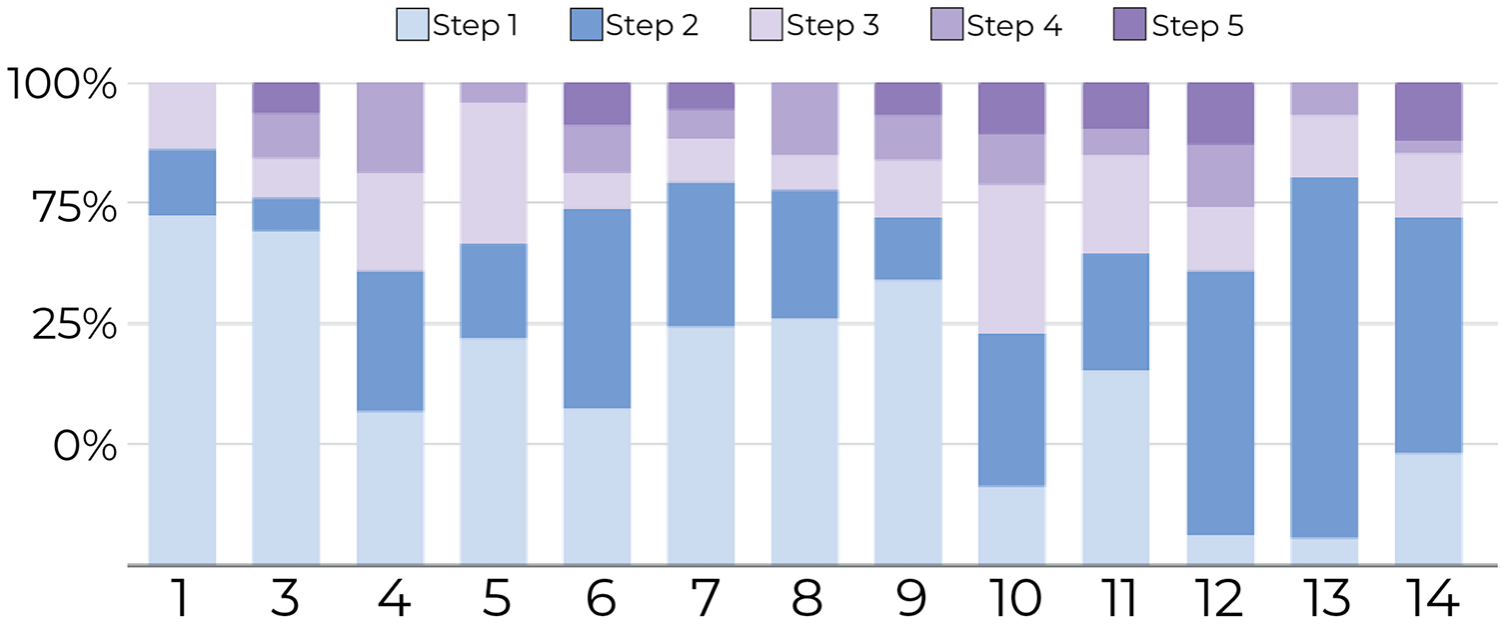
Document movement by study step where steps 1 and 2 are shades blue, and the last three steps are shades of purple. The figure shows that most point movements (y-axis) were made in the initial steps (blue region), and participants (x-axis) tended to move documents less in the later steps (purple region).

**Figure 7:**
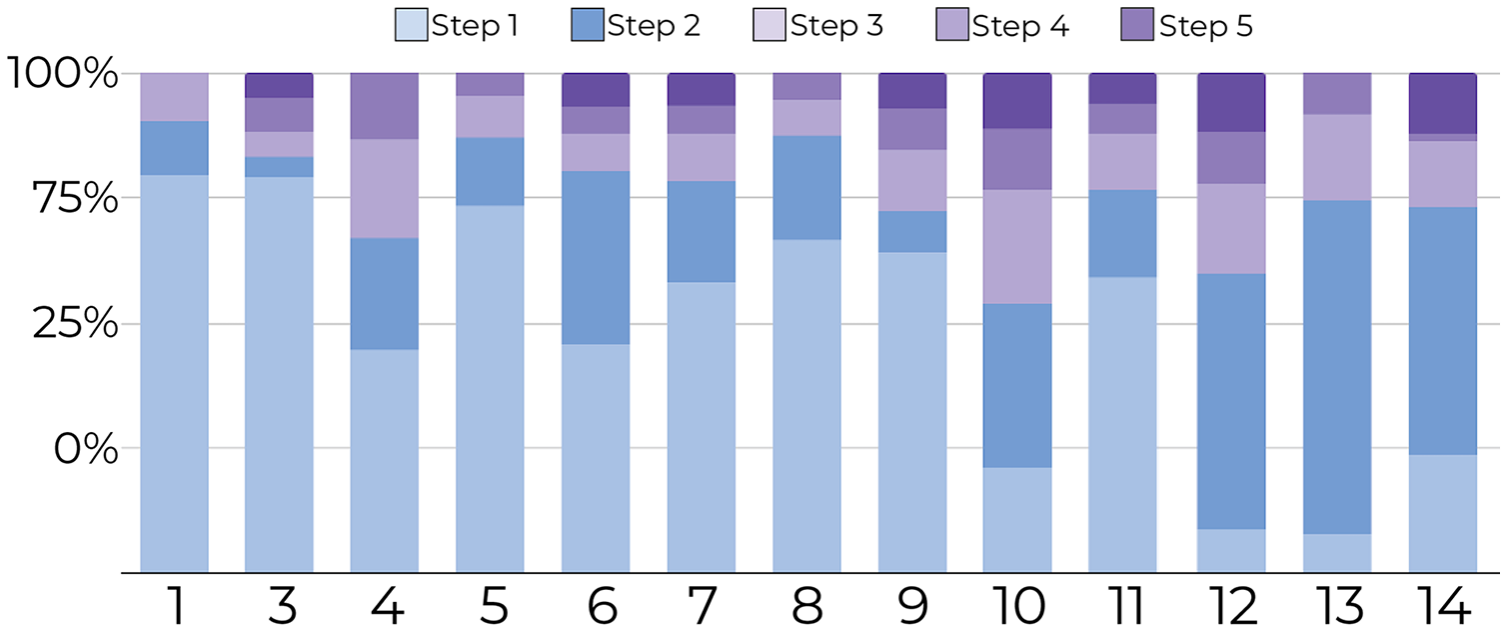
Document movement for only newly introduced documents by study step. Steps 1 and 2 are shades of blue, and the last three steps are shades of purple. For new documents, most movements (y-axis) were performed in the first steps (blue regions) for all participants (x-axis) compared to later steps (purpleregions).

**1-Tracking the number of document movements**: Figures 6 and 7 show the number of movements for all the documents and for only newly introduced documents. Each stacked bar of the chart describes the proportion of document movements made by one of the participants who completed at least 3 steps. In both figures, the number of document movements is decreasing with each step, which indicates that the model becomes better at predicting the appropriate position of a document, thus reducing user intervention. At steps 1 and 5, the average number of movements was respectively 12 and 3.9, suggesting a reduction of 67.5% in the number of movements.

**Figure 8:**
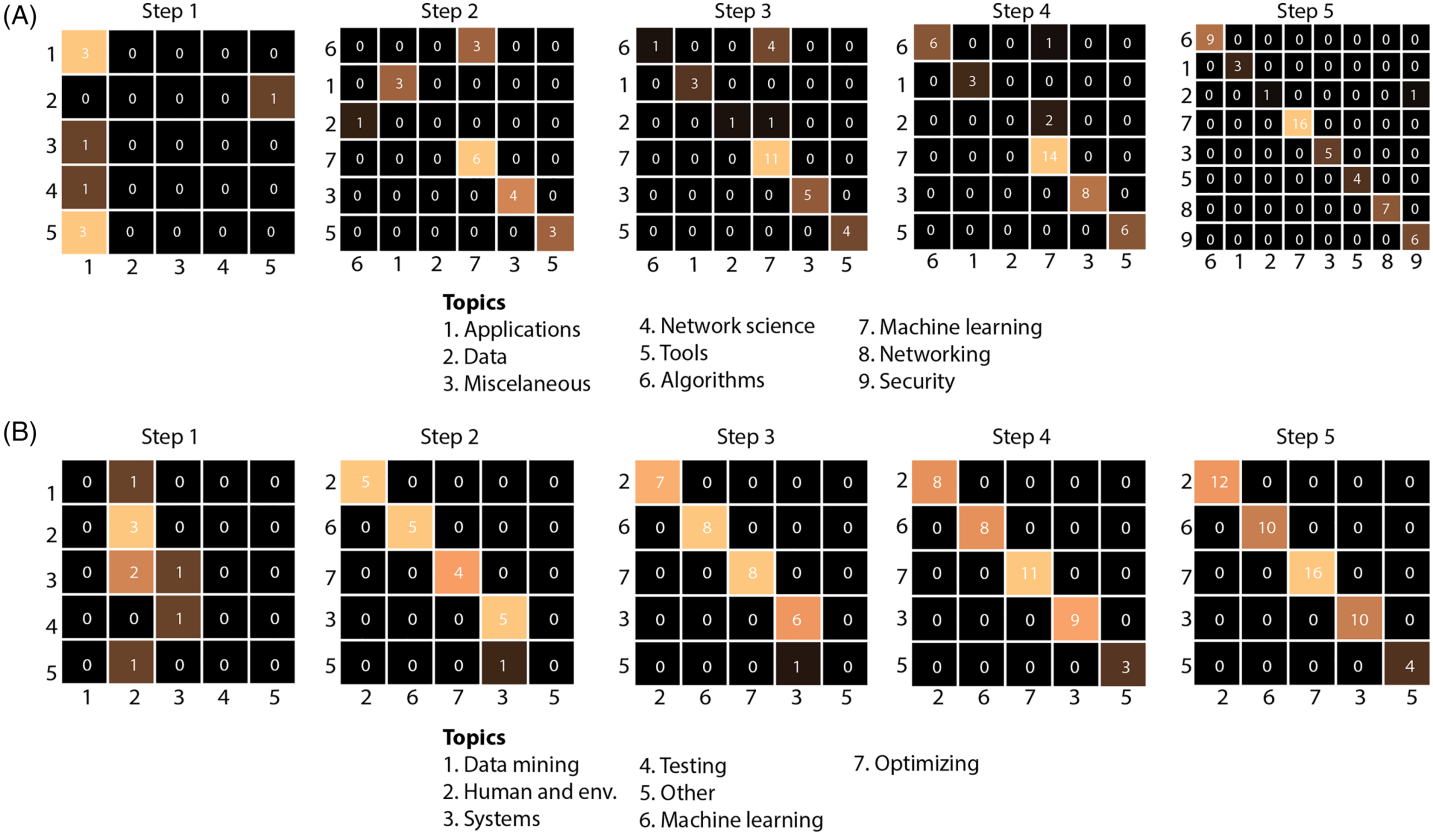
The model’s cluster predictions against the users’ clusters for study participants 11 (A) and 13 (B). The x-axis shows the predicted cluster labels and y-axis shows actual cluster labels for documents introduced at a given step. As illustrated by the brighter cells along the diagonal (higher concordance between predicted and actual labels), the model is increasingly more accurate at predicting the correct cluster as new documents are presented after each step. The complete figure is presented in Fig. S1.

**2-Comparison of model-generated and user-generated cluster predictions:** A comparison of the model’s cluster predictions *vs* the users’ preferred cluster assignments is shown in Fig. 8. This figure is a 2D array of confusion matrices. This confusion matrix shows the number of documents assigned by the model *vs* those assigned by the user (y-axis). Diagonal elements represent documents assigned to the same cluster by the model and user. Figure 8 shows an increasing concordance between predicted and user-assigned clusters with the number of steps, indicating an increasing ability for the model to accurately match users’ preferred placement more closely. A step-wise confusion matrix for all user studies is provided in Fig. S1.

**Figure 9:**
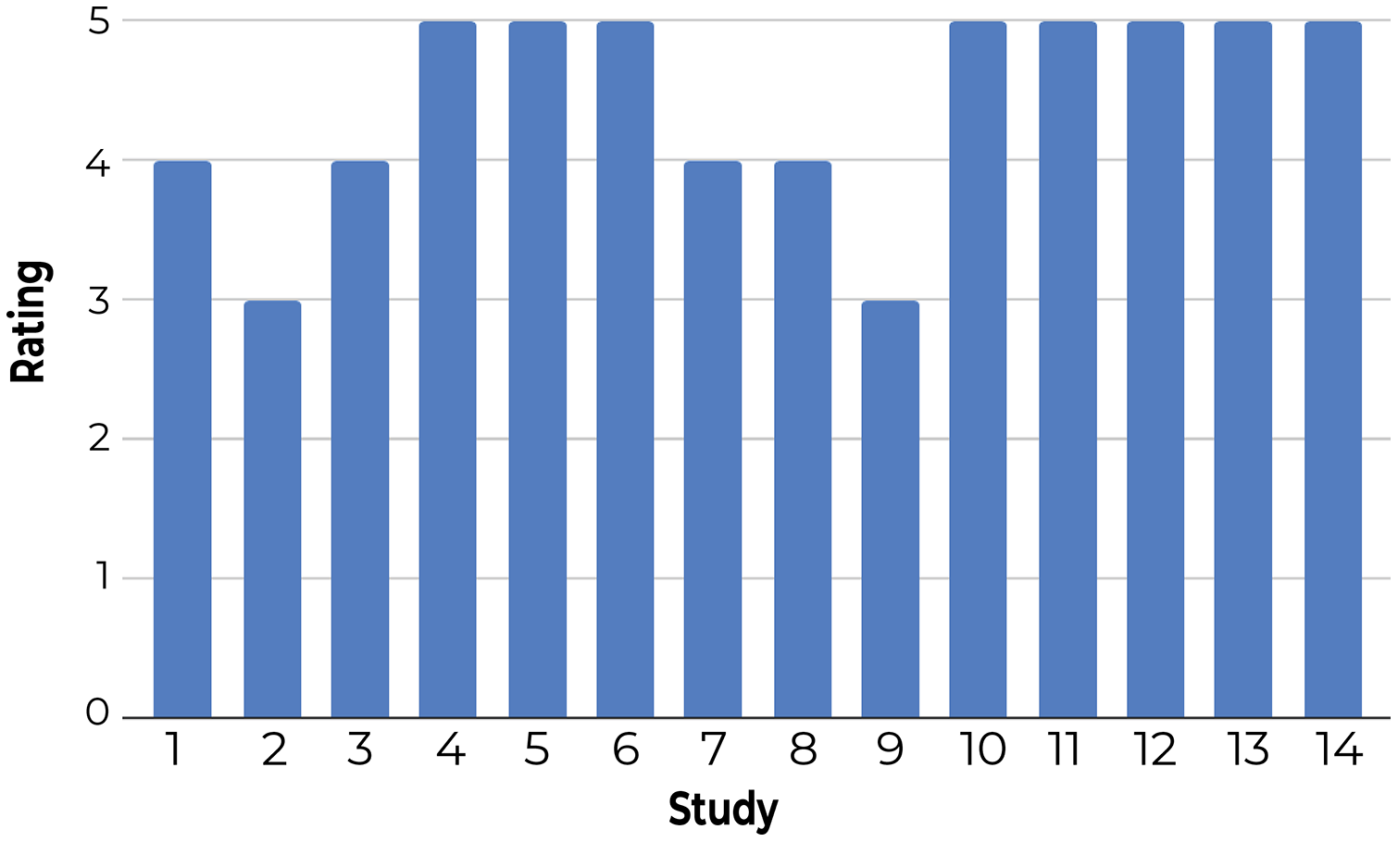
Participants’ average rating of the Z-Explorer system based on the following questions: 1. During each step, how well did the system assign new documents to existing clusters? 2. How well does the model represent how you understand the relationships between these documents? 3. How easy was it to utilize the system to organize these documents?

**Figure 10:**
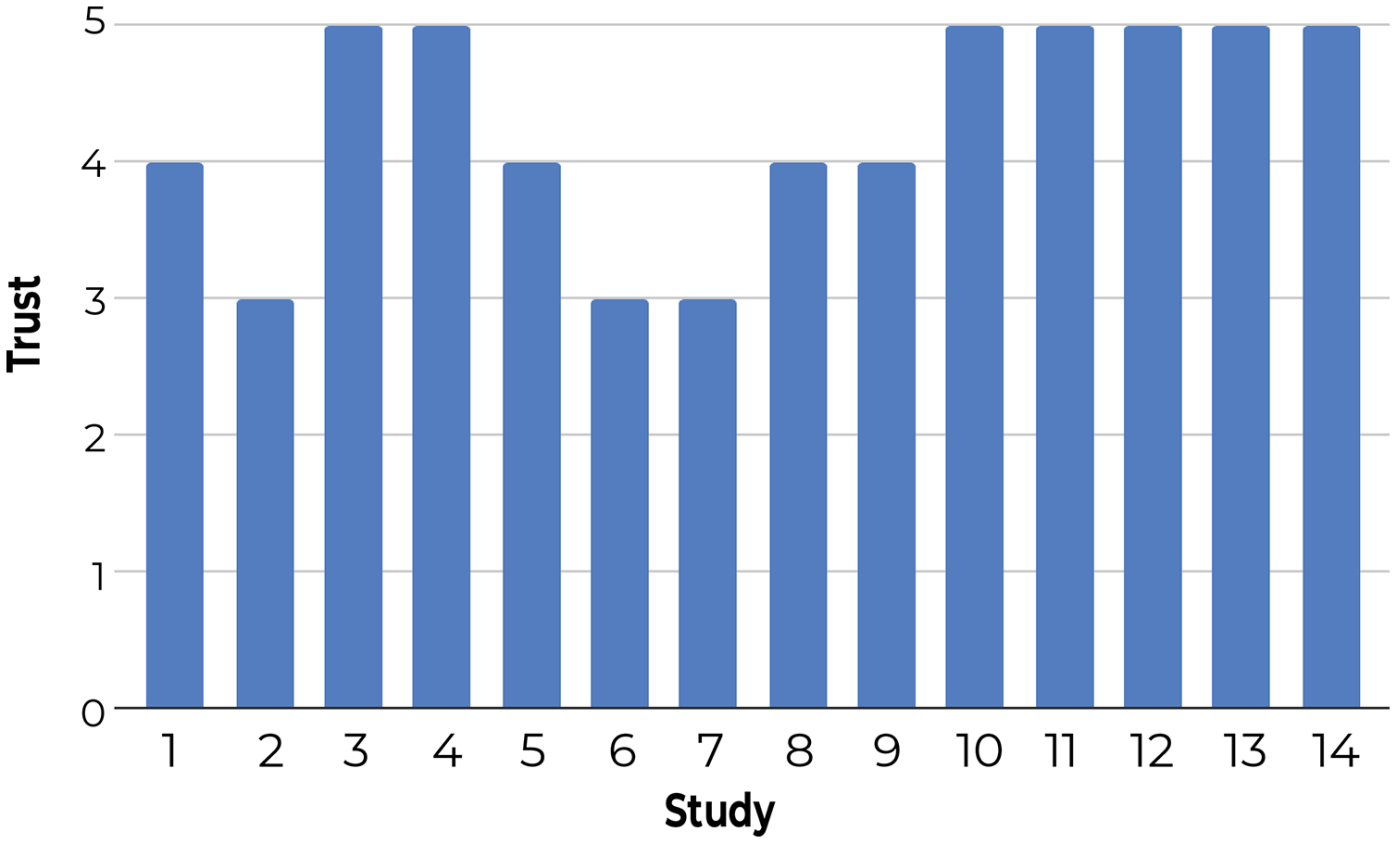
Participants’ average trust rating of the Z-Explorer system based on the following questions: 1. Is the system flexible enough to accommodate changes in your preferences for the grouping of documents? 2. Would you reorganize the document differently in retrospect? 3. Would the system be able to adjust to accommodate alternative ways of organizing the documents?

**3-Using a questionnaire to understand user satisfaction and trust:** Participants rated Z-Explorer based on questions that generally reflect their satisfaction with document positioning (see Fig. 9) and their trust in its reliability and flexibility (Fig. 10). According to both figures, users were satisfied with the Z-Explorer clusters and ease of use (average rating of 4.42 out of 5) and trusted the system could accommodate alternative ways of organizing documents (average rating of 4.28).

### Q2: How does the goodness of clustering compare between Z-Explorer and UMAP in a steerable context?

**Figure 11:**
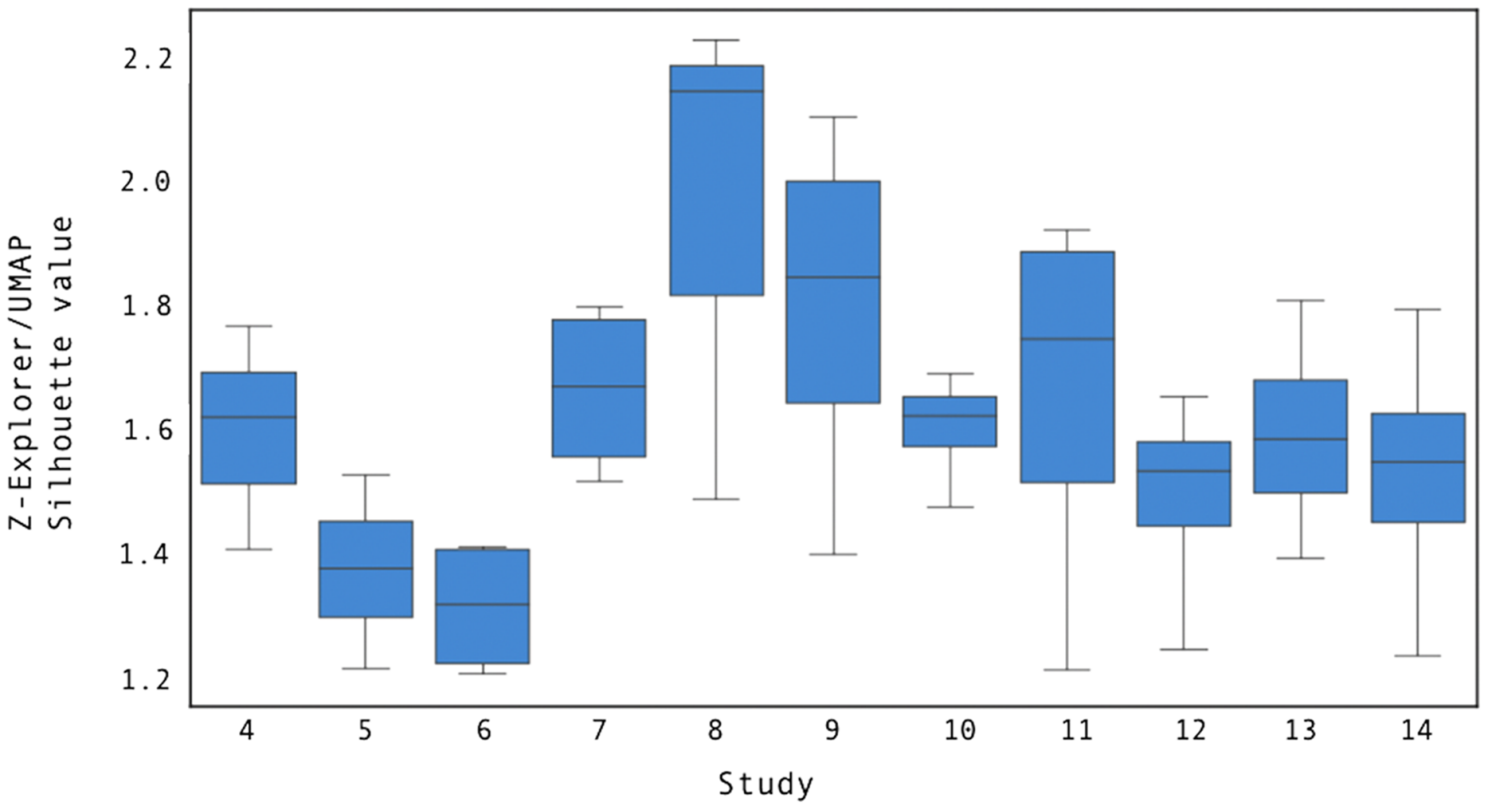
The ratio between the mean Z-Explorer and UMAP silhouette scores for user studies 4 through 14. Each boxplot illustrates the distribution of the silhouette score across all study steps.Compared to UMAP, Z-Explorer improves the goodness of clustering by at least 1.3 times, as measured by the silhouette score.

We compared Z-Explorer and UMAP for their goodness of clustering as measured by silhouette score (Rousseeuw, 1987). At the end of step i-1, we updated the Z-Explorer parameters if any document positions had changed, and we trained de-novo UMAP using the cluster labels that a user had provided to describe the documents on the canvas. Then, in step i, we projected new documents with Z-Explorer and UMAP and calculated their silhouette scores, taking the revised solution given by the user at step i to represent the ground truth. The results of the experiment are summarized in Fig. 11.

As the figure shows, Z-Explorer silhouette scores were at least 1.3 times larger than those obtained by projecting using UMAP. While the precise cause of this cannot be determined precisely, we hypothesize that Z-Explorer benefits from recalling past interactions that resulted in re-parameterization. On the other hand, being *de-novo* retrained at each step *i* with the solution revised by the user at step *i* − 1 (ground truth), UMAP does not retain adjustments through subsequent steps. Analysis of the corrected positions suggested this to be the case. As a consequence of the embedding loss imposed by the model, documents clustered ambiguously by Z-Explorer (*e.g*., placed halfway between two clusters) but later manually moved closer to a specific cluster remained in subsequent interactions close to the cluster to which they had been assigned. However, UMAP failed to maintain prior manual assignments, which contributed to lower silhouette scores.

### Q3 : Do participants create different cluster configurations?

This question was designed to discover whether participants clustered data differently, establishing whether a “one-size-fits-all” approach to clustering is sufficient for organizing documents. This was determined by 1-comparing the number of clusters across participants and 2-assessing the concordance between clusters to determine if differences in the number of clusters were restricted to a few documents, or if they were substantial.

**Figure 12:**
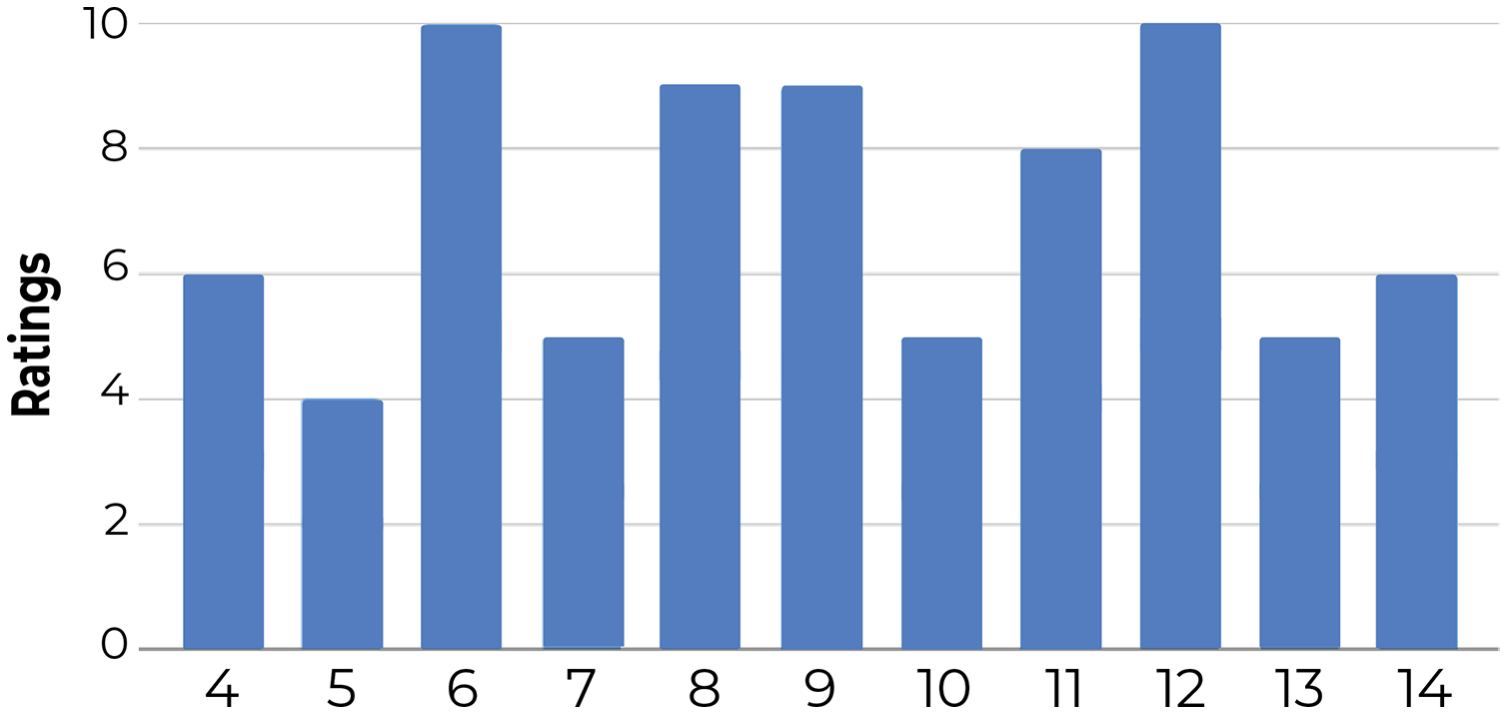
Number of clusters created by users that completed all five steps and reviewed all 52 documents. The results show that the number of clusters vary across participants, ranging between four in Participant 4 and 10 for Participants 6 and 12.

**1-Comparing the number of clusters across participants**: Figure 12 shows that the number of clusters created by participants varied between four and 10 (for a fair comparison, we only include the results of 11 participants who examined all 52 documents). This confirms that the clustering solutions diverged at least in the number of clusters.

**Figure 13:**
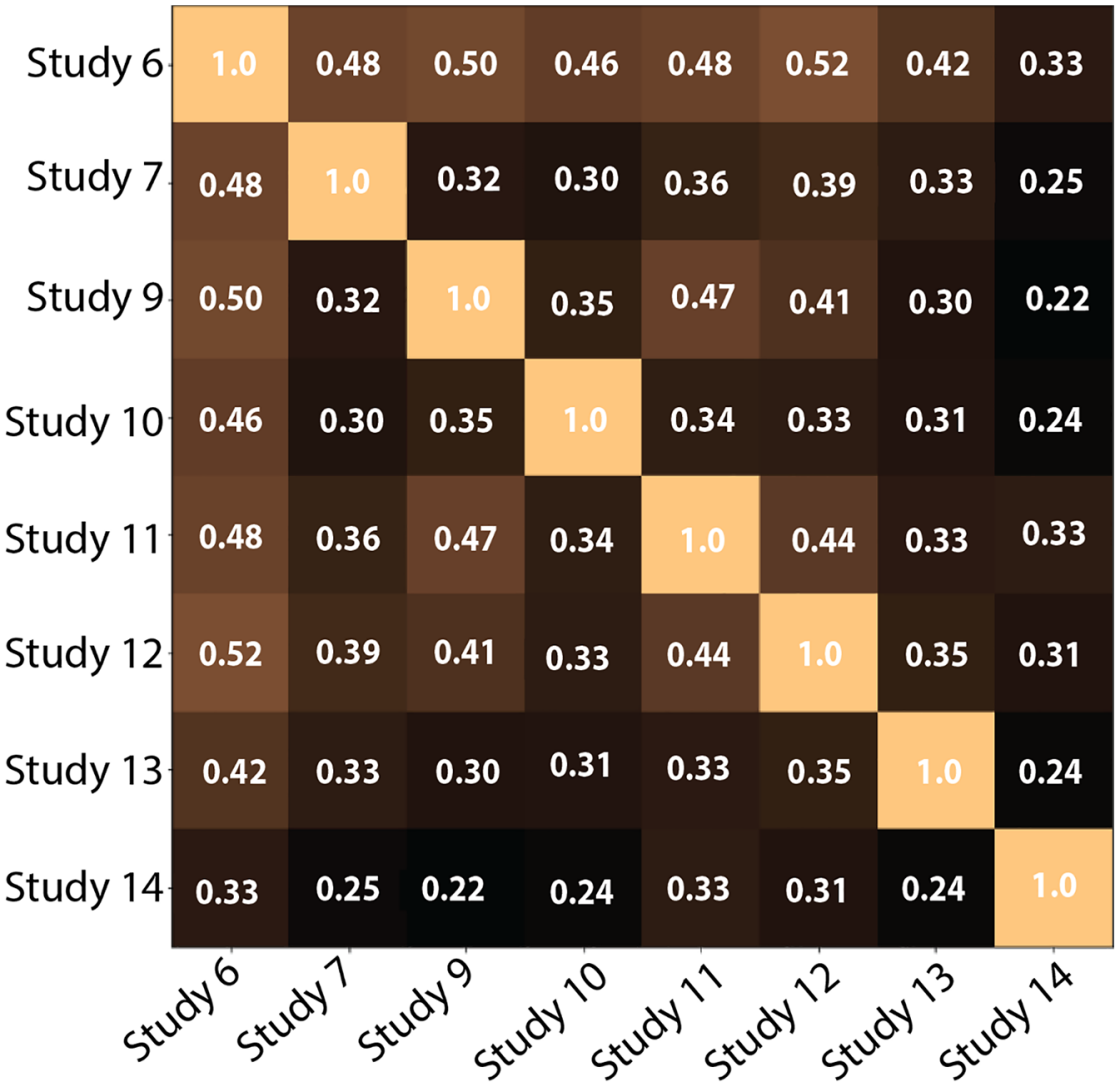
Pairwise V-Measure-score across eight studies (Participants who examined all 52 documents and for whom the document-to-cluster assignments were recorded). Lighter colors depict higher scores. Diagonal shows a perfect score when comparing a study to itself. As seen in the figure, the value is lower than 0.5 for most of the cells (darker cells), which correlates with our hypothesis that the participants did create different clusters.

**2-Comparing the number of clusters across participants**: Figure 13 shows the pairwise V-Measures, which determine the homogeneity of clusters across different studies; as previously stated, those were the users who reviewed all 52 documents and for whom we successfully stored the model outputs. The V-Measure scores were all computed with *β* = 1.0, which weighs homogeneity and completeness equally. V-Measure values range from 0 (no similarity) to 1 (identical clusters). In the figure, the value for most cells is below 0.5, suggesting that participants clustered the documents differently.

The results confirm that participants created different clusters, both in number and in the degree of overlap, to organize the same dataset. This emphasizes the importance of semantic interaction and the inadequacy of a one-size-fits-all solution for clustering documents.

The target Euclidean distance (*c*_*p*_) utilized in our user studies was computed from documents placed at random on the canvas. The approach is ideal for small datasets since it leaves it up to the users to decide the initial layout that will be refined in future iterations. However, manually producing an initial setup may be labor-intensive with large datasets. Thus, it may be worthwhile to precompute an initial layout, using for instance, UMAP to produce a 2D representation of the data. Whether the initial placement is random or precomputed using UMAP, users are likely to rearrange the data as they see fit, so the only advantage of the precomputed layout remains convenience.

Lastly, the model proposed here is relatively small (approximately 200 k parameters) when compared to models such as DeepSI (Bian & North, 2021b), which fine-tune BERT base model (110 M parameters), and can be trained using a subset of the data (*e.g.*, randomly selected pairs of documents), thus reducing training complexity and enabling close to real-time processing speeds needed for such human-in-the-loop systems (Zhang et al., 2021).

## Conclusion

This paper proposes a new technique for enhancing semantic interaction in visual analytics systems using deep learning. Our approach provides a computationally efficient surrogate deep contrastive learning model for the dimensionality reduction problem *via* semantic interaction. By doing so, it enables non-expert users to tune this flexible deep learning model, thus conveying their beliefs, preferences, or domain expertise about data organization by simply rearranging the data in a lower-dimensional representation. Our research shows that the properties of the deep contrastive learning model, together with our proposed parametric feedback for updating model parameters based on user interaction, make the proposed model a viable solution for the SI pipeline.

We provide evidence of the efficacy and utility of our approach by:

1. Developing a proof-of-concept system that illustrates how a contrastive learning-based model can be used to support SI.
2. Demonstrate through a user-study that the SI + DCL paradigm can capture user-specific cluster preferences with fewer steps than manual clustering of the data, thus underscoring the necessity and value of incorporating user feedback in AI-enhanced analytics applications.
3. Confirming that the approach can be usefully integrated into an application, namely Z-Explorer- a visual document analysis add-on tool for Zotero.

Although Z-Explorer, our example application, focused on text document analytics, our approach can be broadly applied to other data types and applications that value involving the user in the loop during an analytical process.

## Deep Learning Model

### Model architecture

Architecture of the neural network. Each of the twin networks $(F^{(1)}_W {\rm and}\ F^{(2)}_W)$ consists of four dense layers (*D*1 through *D*4) and a final embedding layer $E$. Each dense layer computes the forward pass, where the input is the output of the previous dense layer. The layers of these twin networks share their weights and implement the same parameterized function. Each twin network transforms the inputs into 2D embeddings fed to the $k$-dimensional softmax layers to yield cluster predictions. Additionally, the predicted contrastive distance is computed on the output projections. These aforementioned outputs are then fed to the three loss heads of the model.

### Model training

After reorganizing the visual space, the user submits the newly labeled dataset to fine-tune the model inferred from previous steps. The new training data is selected to consist of a small subset of input pairs that were randomly selected from the newly labeled dataset. Given that in each iteration, users update the positions of a relatively small number of documents, we have used a fine-tuning training approach to allow models to retain the weight learned from previous steps. In our tests, we found that fine-tuning the model instead of training *de-novo* reduced training time, minimized over-fitting, and improved performance.

The contrastive model was developed using the Python Keras deep learning framework. Each fully connected layer $D_l$ of Fig. 2 in the model utilizes dropout and batch normalization to minimize overfitting, and ensure that parameter tuning is made as efficient as possible. In our tests, we observed that training the model on 30% of all possible pairs over 10 epochs using the Adam optimizer with a learning rate of $\eta$ = 0.1 provided the best results. This large learning rate was chosen to compensate for the relatively small number of epochs. Model implementation details and default parameters are available in the ‘encoder.py’ file which can be found in the project GitHub repository.

### Effective number of study participants

Unfortunately, two participants ran out of time and could not complete more than two steps. As such, we decided to exclude the results of participants who did not complete at least three steps to ensure the results were fairly comparable across participants. During the course of the study, we realized that it may be useful to record Z-Explorer’s predicted cluster assignment for each document and compare it to the participants’ assignments. However, by then, we had already completed six studies, and, therefore, were only able to capture this data for nine participants. We nevertheless present the results of those nine participants as they do yield insightful results (Fig. 8).

## Supplemental Information

10.7717/peerj-cs.925/supp-1 Supplemental Information 1 Results from User Studies.

10.7717/peerj-cs.925/supp-2 Supplemental Information 2 Aggregated data from the user study.

## Additional Information and Declarations

### Data Availability

The following information was supplied regarding data availability: The code is available on GitHub: https://github.com/whatwehaveunlearned/zExplorer.
