# Supplementary material for: Leveraging deep contrastive learning for semantic interaction
Source: PeerJ Comput Sci. 2022 Apr 8;8:e925. doi: 10.7717/peerj-cs.925 (PMC9044347; doi:10.7717/peerj-cs.925)
Supplement: Supplemental Information 1 [file peerj-cs-08-925-s001.pdf]

1 SUPPLEMENTARY FIGURES

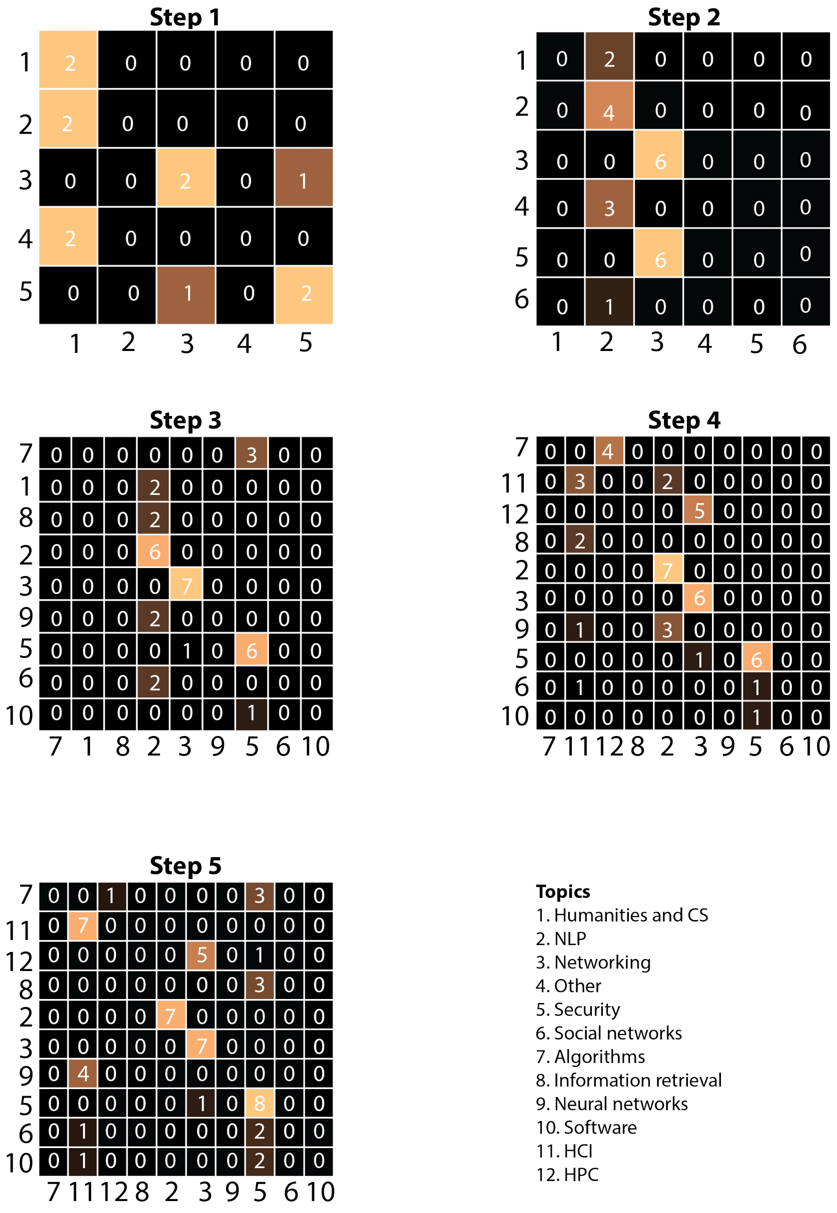

Figure 1. User Study 6.

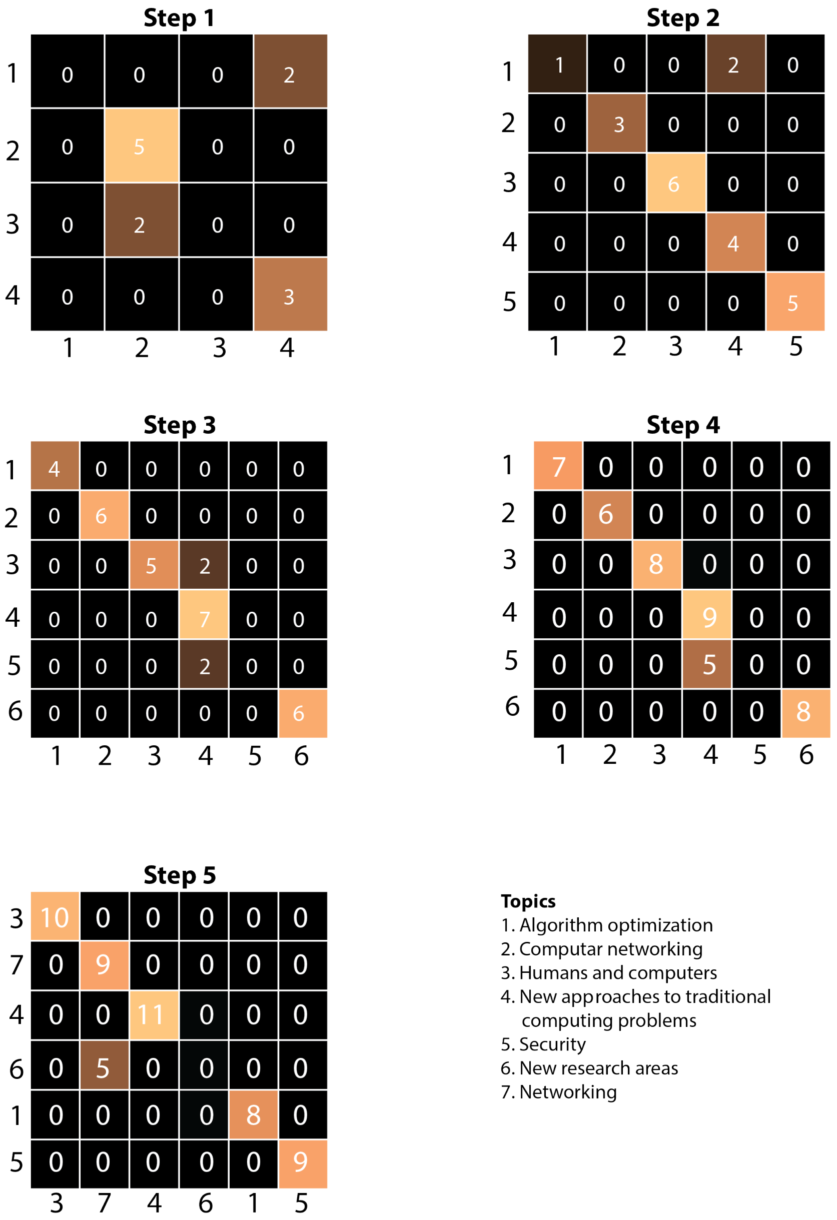

Figure 2. User Study 7.

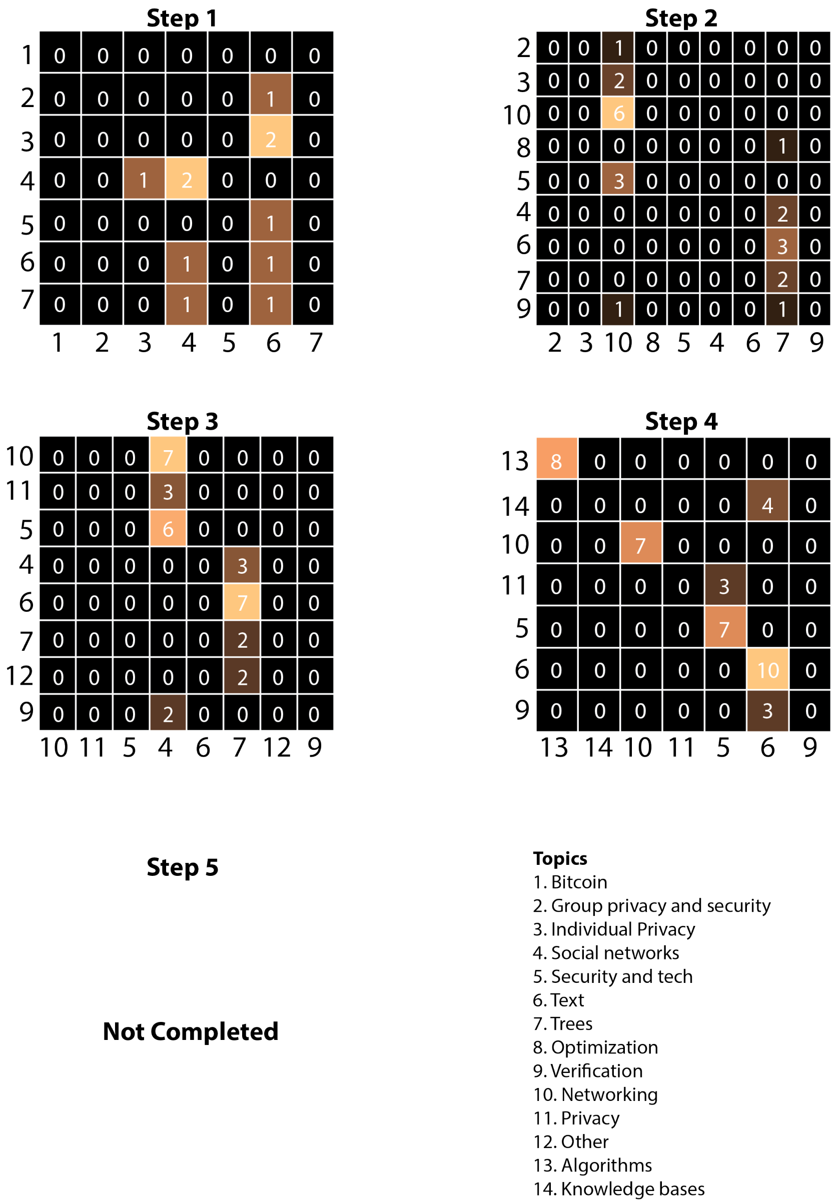

Figure 3. User Study 8.

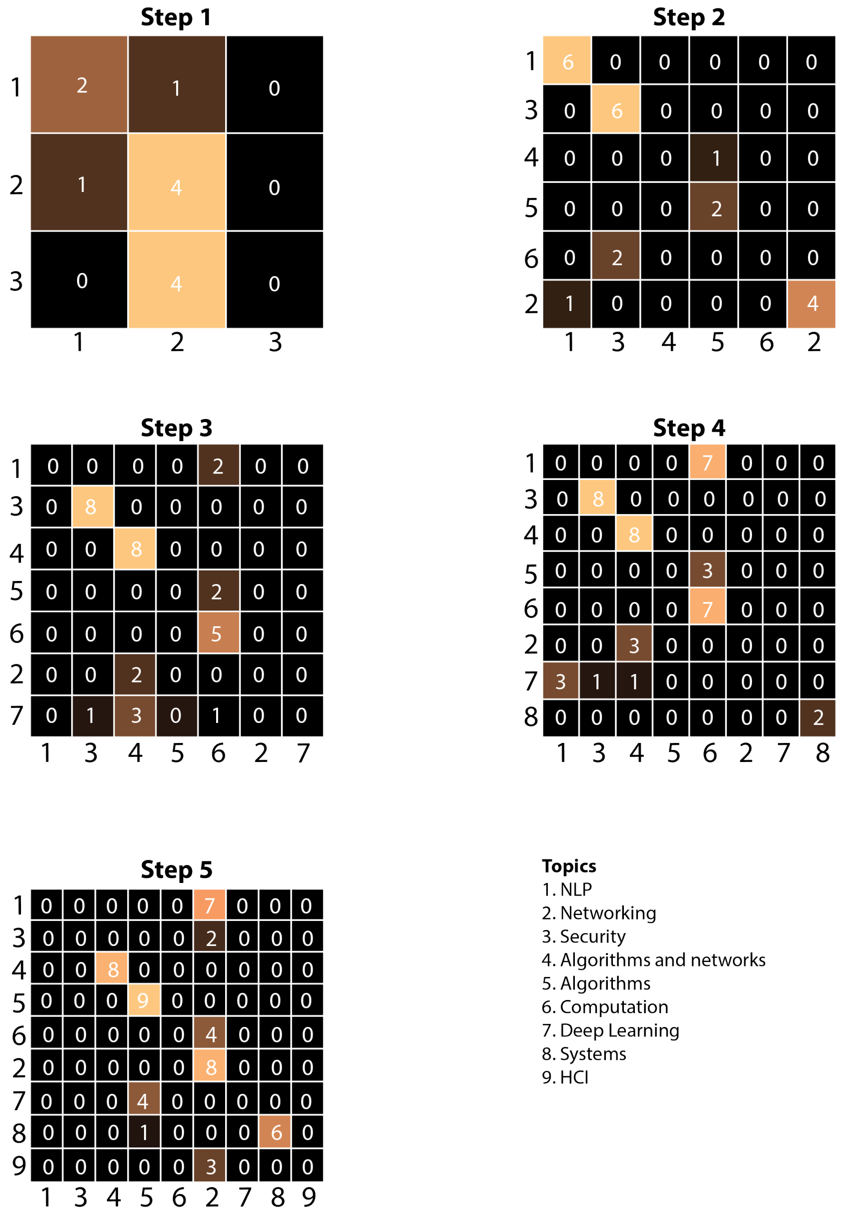

Figure 4. Use Study 9.

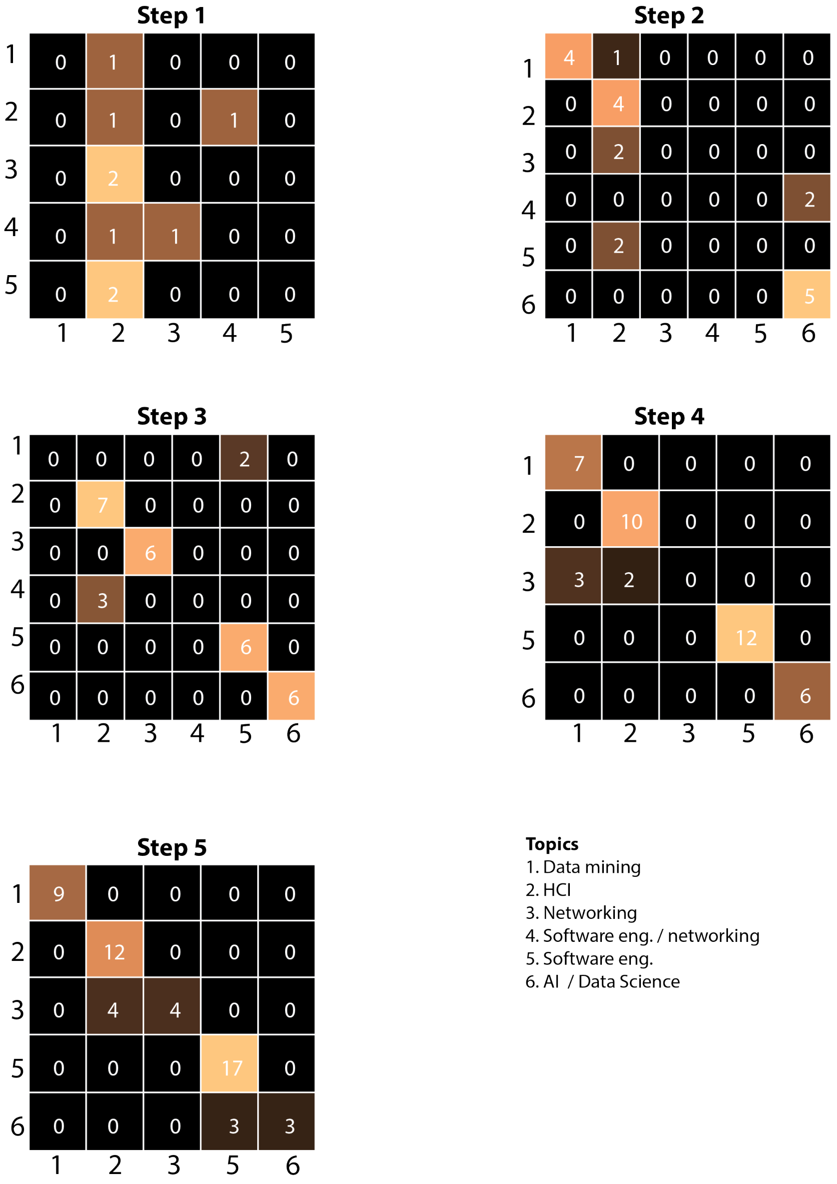

Figure 5. User Study 10.

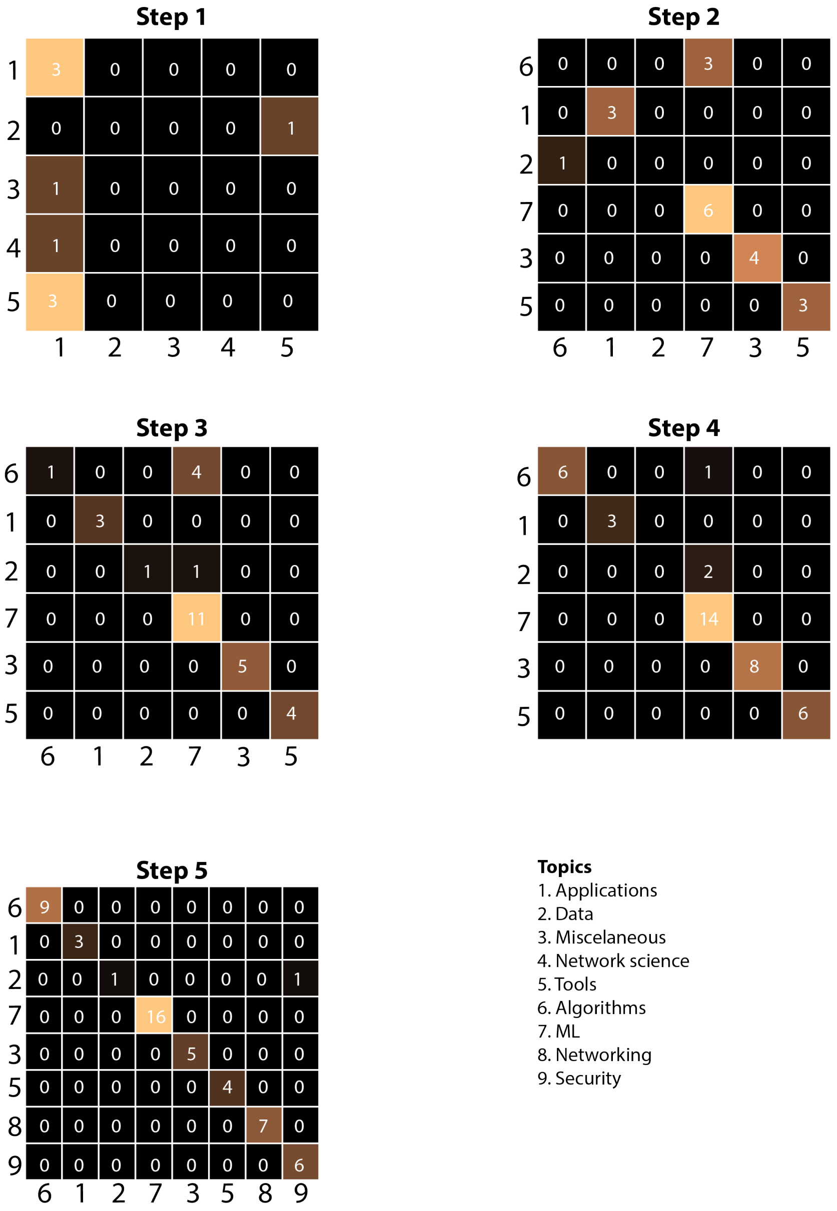

Figure 6. User Study 11.

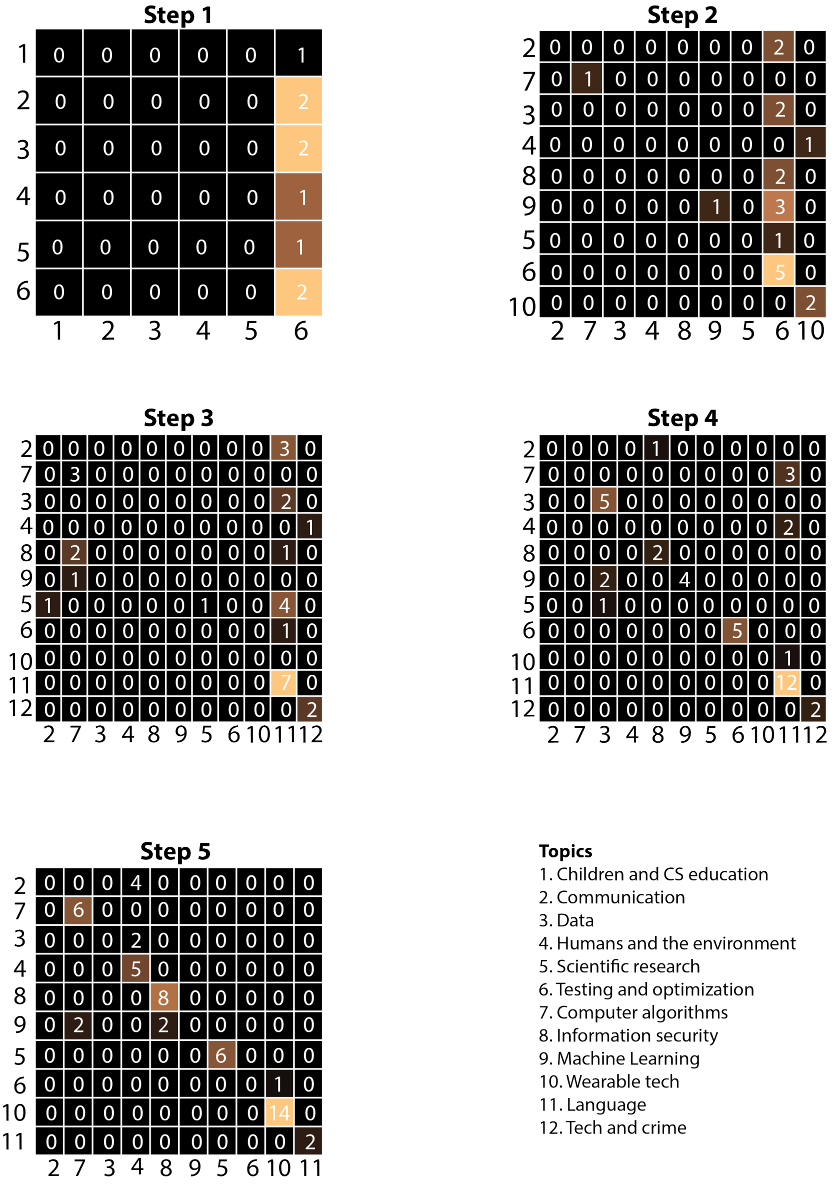

Figure 7. User Study 12.

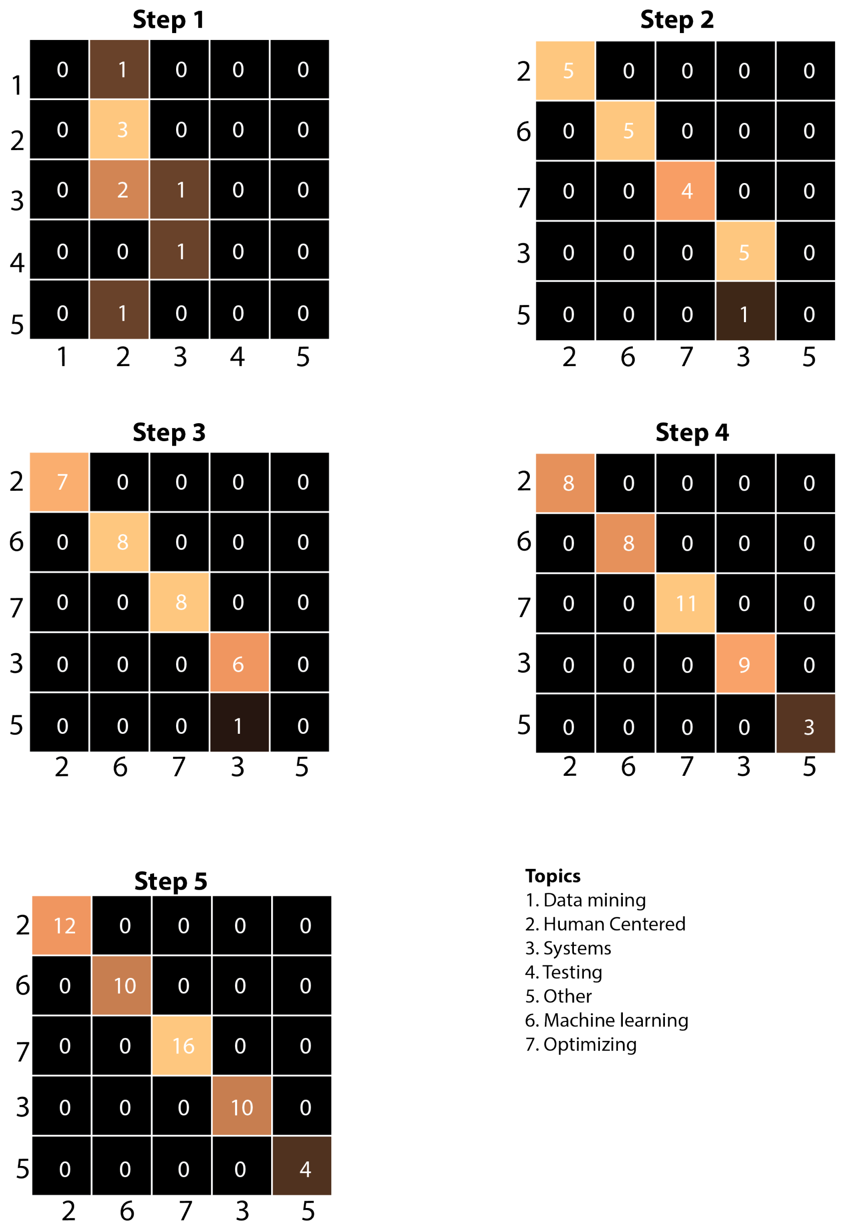

Figure 8. User Study 13.

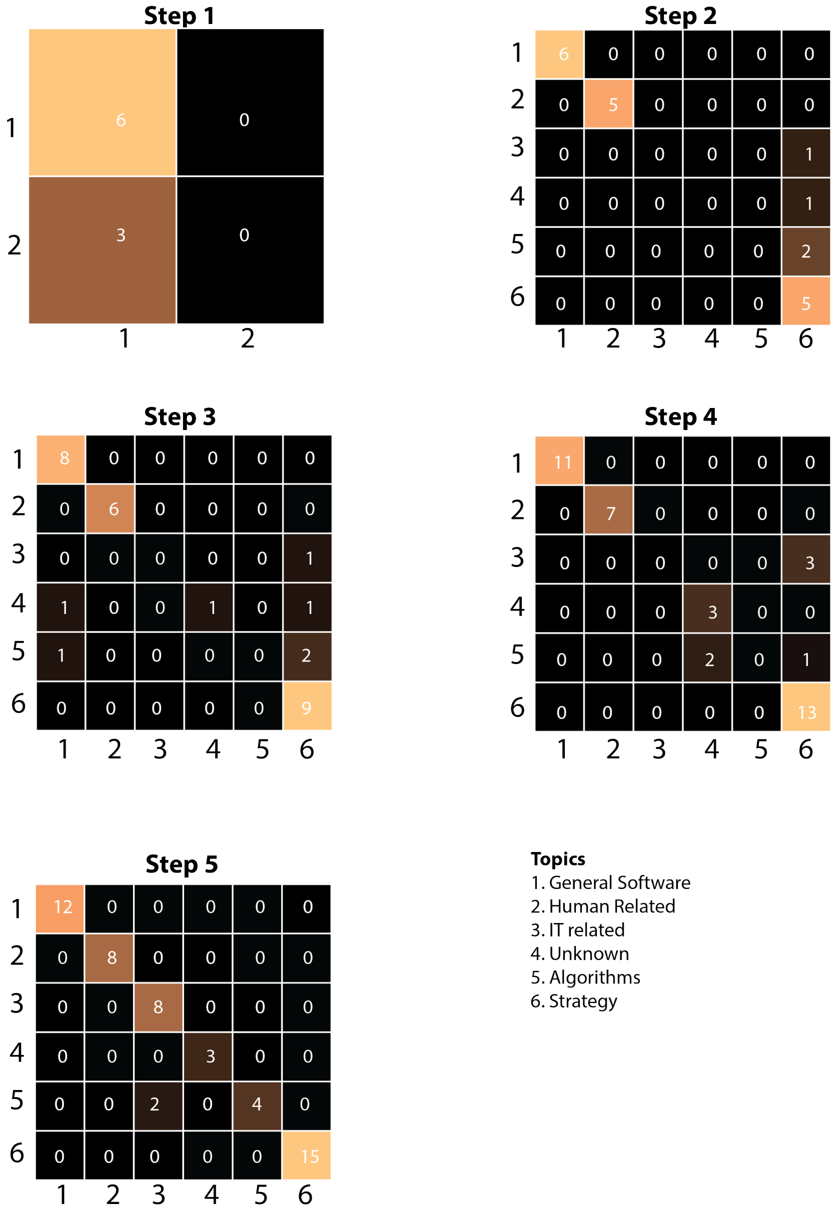

Figure 9. User Study 14.
