# Supplementary material for: Leveraging deep contrastive learning for semantic interaction
Source: PeerJ Comput Sci. 2022 Apr 8;8:e925. doi: 10.7717/peerj-cs.925 (PMC9044347; doi:10.7717/peerj-cs.925)
Supplement: Supplemental Information 2 [file peerj-cs-08-925-s002.zip › studies_data/Documents Manifest.docx]

- **cluster_sumaries_by_study_and_step.xlsx:** Each line in this file lists the cluster name, as provided by the user, for each step (column) of the iterative algorithm
- **summary_data.xlsx:** Summaries the data across all experiments for all users.
  - **Num doc per study:** Number of documents at each step of each study.
  - **Distance moved (all points):** Shows the sum the distance by which each document was moved in each study.
  - **Distance moved (new points):** Shows the sum the distance by which each document was moved in each study.
  - **Number documents moved (new points):** Nummer of new documents moved at each step.
  - **Number of documents not moved:** Shows the number of new documents that were not moved at each step.
  - **Number of clusters:** Number of clusters at each step for each study.
  - **Time per step:** Shows the time spent at each step for each study
  - **Questions per study compilation:** Shows answers recorded for studies to the open questions asked. The bottom section shows the participant’s ratings for the system at the end of the experiment.

The studies are summaries in documents **summary_1.xlsx, … summary_14.xlsx.** The content of each document is described below:

- **Study1/summary_1.xlsx:** Summarizes the results of each study participant. The file contains the following columns, where *i* represent the step number:
  - **Key:** The publication unique identifier
  - **Title:** The publication title
  - **Cluster i**: Cluster assigned to this document by the participant at step i
  - **Distance_moved i:** Distance that the participant moved the document in step i.
  - **x i**: Position along the X-axis for the document at step i
  - **y i**: Position along the Y-axis for the document at step i

For user who completed all five steps, this document contains five repetitions of the columns
